# Relationship between psychosocial stress and hypertension among Ghanaians in Amsterdam, the Netherlands – the GHAIA study

**DOI:** 10.1186/1471-2458-14-692

**Published:** 2014-07-07

**Authors:** Bernard Agyei, Mary Nicolaou, Linda Boateng, Henriette Dijkshoorn, Bert-Jan van den Born, Charles Agyemang

**Affiliations:** 1 Department of Public Health, Academic Medical Centre, University of Amsterdam, Meibergdreef 9, 1105, AZ Amsterdam, The Netherlands; 2 Department of Epidemiology, Documentation and Health Promotion, Public Health Service Amsterdam, Amsterdam, The Netherlands; 3 Department of Internal Medicine, Academic Medical Centre, University of Amsterdam, Meibergdreef 9, 1105, AZ Amsterdam, The Netherlands

**Keywords:** Psychosocial stress, Hypertension, Ethnicity, Migration and health

## Abstract

**Background:**

Hypertension is highly prevalent among recent sub-Saharan African (SSA) migrants in western countries and some tend to associate their hypertension to psychosocial stress. However data on the relationship between hypertension and psychosocial stress among SSA migrants are rare. We assessed the relationship between psychosocial stress and hypertension among the largest SSA migrant population (Ghanaians) in Amsterdam, the Netherlands.

**Methods:**

Data were obtained from structured interviews along with medical examination among 212 participants from a cross-sectional study: the GHAIA study in 2010 in Amsterdam. Blood pressure was measured with a validated Oscillometric automated digital blood pressure device. Psychosocial stress was assessed by questionnaires on perceived discrimination, depressive symptoms and financial problems. Binary logistic regression was used to study associations between psychosocial stress and hypertension.

**Results:**

The overall prevalence of hypertension was 54.7%*.* About two thirds of the study population experienced a moderate (31%) or high (36%) level of discrimination. 20.0% of the participants had mild depressive symptoms, whilst 9% had moderate depressive symptoms. The prevalence of financial stress was 34.8%. The psychosocial stresses we assessed were not significantly associated with hypertension: adjusted odds ratios comparing those with low levels and those with high levels were 0.99 (95% CI, 0.47–2.08) for perceived discrimination, 0.81 (95% CI, 0.26–2.49) for depressive symptoms and 0.71 (95% CI, 0.37–1.36) for financial stress, respectively.

**Conclusion:**

We did not find evidence for the association between psychosocial stress and hypertension among recent SSA migrants. More efforts are needed to unravel other potential factors that may underlie the high prevalence of hypertension among these populations.

## Background

Many ethnic minority groups in Western countries, particularly sub-Saharan African (SSA) origin (henceforth, African origin) populations are disproportionately affected by hypertension [1-5]. In the Netherlands, for example, the prevalence of hypertension is twice as high among African-Surinamese men, and nearly four times higher among African-Surinamese women than among White Dutch men and women [5]. Complications of hypertension [1] are also higher in African origin people than among populations of European origin (henceforth, Whites). For example, people of African origin have a relatively higher risk of stroke and end-stage renal failure than White people [1,6].

Hypertension is also becoming a public health burden in the SSA region [7]. However, the prevalence rates in SSA countries are much lower than those reported among SSA origin populations living in Europe [4,5] and North America [2].

The high prevalence of hypertension among SSA origin populations in Western countries is difficult to explain given the absence of data on the relative importance of environmental and genetic factors. Several explanatory factors including environmental and genetic factors have been proposed [4,8]. Stress is among the psychological variables that has long been listed among the potential and important risk factors of hypertension and coronary heart disease [9,10]. Stress has been defined as a process in which environmental demands exceed the adaptive capacity of an organism. This can result in psychological and biological changes that may place persons at risk of disease [11]. There is large variability of exposure and outcomes in terms of stresses and the subsequent effects. Production of stress hormones, for example cortisol, may be triggered by normal physiological activities in humans. This may help prepare the individual sensing or involved in a stressful situation [12]. However, prolonged activation of stress hormones can be harmful. The mechanisms underlying the association between psychosocial stress and hypertension can be divided into behavioural and pathophysiological mechanisms. The former contributes to adverse health behaviours such as physical inactivity, poor diet and smoking, whilst the latter involves neuro-endocrine activation mediated by the hypothalamo-pituitary-adrenal (HPA) system [12,13]. Several studies have supported these underlying mechanisms which may lead to sustained elevated blood pressure, but others do not [14]. Furthermore, acute stressful events have no consistent association with hypertension. Chronic stress on the other hand, particularly the non-adaptive response to stress, have been reported as more likely the cause of sustained elevation of blood pressure [15].

Migration has been viewed as a highly stressful process and migrants are very vulnerable to mental disturbances. A number of studies have explored the prevalence of mental illness in different migrant groups. In many of these studies mental health problems including psychosocial stress are much more common among migrants than among non-migrants [16-18]. In the recently migrated SSA populations where many leave their native countries for the Western world due to political reasons and or economic hardships [17,19], psychosocial stress and other mental health problems can also be highly prevalent. Furthermore, adapting to a new environment, difficulty finding a suitable job or earning a living abroad can be very challenging. This may result in psychological distress for many migrants, particularly those with undocumented status [16,17]. As a result, some African migrant groups associate hypertension with migration related stress. In a previous qualitative study in the Netherlands among hypertensive Ghanaian, African Surinamese and Dutch patients, many Ghanaian and African-Surinamese respondents attributed hypertension to migration-related factors. Some of these factors were stress owing to difficulties in adapting to the much demanding Dutch society and obligations towards their families in their homelands. The latter was much more reported among Ghanaian respondents, a relatively new migrant population in the Netherlands [20,21]. Also in prior studies among Ghanaian migrants in Amsterdam, financial strain both abroad and from the homeland, economic hardship and discrimination were reported as some of the problems this migrant group faces [17]. Perceived daily exposure to racial discrimination can form a chronic stressor, which can affect an individual’s physical health. This together with other migration related stresses could result in stress related diseases such as (symptoms of) depression or emotional distress. Also factors like the strain of adaptation and integration and lack of one’s ability to resist this strain, sometimes in harsh social conditions in the host country may all play a role. This can also result in adverse health behaviours, inadequate coping and dysregulation of stress hormone production in the body which may contribute to the high prevalence of hypertension reported among some immigrant populations in Western countries.

The literature shows inconsistent results on the relationship between psychosocial stress and hypertension [13,14,22,23]. In addition, little is known about the relationship between psychosocial stress and hypertension among the recently, migrated SSA populations living in Western countries. The main objective of this study was therefore to assess the relationship between psychosocial stress and hypertension among Ghanaians, a recent SSA migrant population in Amsterdam, the Netherlands.

## Methods

Data were obtained from the GHAIA study. The full details of the study methods have been published in our previous reports [24,25]. In brief, the GHAIA study (acronym for: Ghanaians in Amsterdam) was a cross-sectional study that comprised of structured interviews along with a brief medical examination among Ghanaian migrants in 2010 in Amsterdam, the Netherlands. Ghanaians are the largest SSA migrant population living in Amsterdam. The study was based on a sample of 18–65 year old Ghanaians in Amsterdam who were not in residential care. The participants (both documented status and undocumented status) were drawn from six Ghanaian churches and a community organization within the Ghanaian Muslim community. In total 221 people participated in the interview of which 212 participated in medical examination. Health behaviour, health conditions and medication use were among the data collected during participants’ interview. Anthropometrics and blood pressure were among the measurements taken during the medical examination. The Medical Ethical Committee of the Amsterdam Academic Medical Centre approved the study protocols (MEC 10/054). Inform written consent was obtained from all participants involved in the study.

### Dependent variable

Hypertension was the outcome variable and was defined as systolic blood pressure ≥ 140 mmHg, or diastolic blood pressure ≥ 90 mmHg, or being on anti-hypertensive medication. Systolic hypertension was defined as systolic blood pressure ≥ 140 mmHg, and diastolic hypertension was defined as diastolic blood pressure ≥ 90 mmHg. Blood pressure was measured in the morning with a validated oscillometric automated digital blood pressure device (OMRON M–6). Using appropriate cuff sizes, three readings were taken on the right arm in a seated position after the subject had emptied their bladder and had been seated for at least 5 minutes. The mean of the last two readings was used in the analyses.

### Main independent variables

Psychosocial stress was the main independent variable and it was defined as perceived discrimination, depressive symptoms and or financial stress. Psychosocial stress was assessed through standardized and validated questions on discrimination and depressive symptoms and whether participants had problems paying their household bills (henceforth, financial stress). We focused on these three factors as some of the major contributors of psychosocial stress among Ghanaian migrants in Amsterdam because Ghanaian migrants reported experiencing financial problems and discrimination in some previous studies [17]. Furthermore, symptoms of depression are among the commonly reported symptoms, which have been associated with psychosocial stress [15]. Depressive symptoms can be viewed as more or less a result of psychosocial stress or a loss, mediated by the ability of an individual to cope with the stresses or losses and the perceived social support. Migrant populations may be more susceptible to these stresses partly due to social, economic and political marginalization.

Perceived discrimination was assessed through a questionnaire about participants own experience of discrimination using the validated Everyday Discrimination Scale by Forman *et al.*[26]. The Everyday Discrimination Scale is a widely used measure of daily perceived discrimination. The questions were about things that may have happened in participants day to day life that they think might have something to do with their ethnicity or colour of their skin. Questions were asked about 9 items: you are treated with (i) less politeness, (ii) less respect, (iii) you received poorer service than other people, people act as if they (iv) think you are not smart, (v) are afraid of you, (vi) you are dishonest, (vii) are better than you, (viii) you are called names and (ix) threatened or harassed. Participants could choose from the following alternatives (with their respective scores in brackets) according to how often the aforementioned events have happened to them; never (1), hardly ever (2), not too often (3), fairly often (4) and very often (5). The total sum of the scores was calculated for each participant. The scores were subdivided into 3 equal groups with their respective cut-off points; ≤ 12.66 (tertile 1), 12.67 – 16.99 (tertile 2) and ≥ 17.00 (tertile 3). Perceived discrimination was classified into 3 levels accordingly: the lowest tertile was considered low level of perceived discrimination, whilst the middle and the highest tertiles were considered moderate and high level of perceived discrimination respectively.

The Patient Health Questionnaire (PHQ-9) [27], a depression diagnostic and severity measure was used as a tool to assess the level of depressive symptoms among the participants. A higher score indicates a higher depression severity. Depressive symptoms was first classified into 5 categories namely minimal, mild, moderate, moderately severe and severe depressive symptoms according to the PHQ-9 depression severity criteria. Respondents with scores below 5 were classified as having minimal depressive symptoms, those with a score range of 5–9 as having mild depressive symptoms, those with a score range of 10–14 as having moderate depressive symptoms, those with a score range of 15–19 as having moderately severe depressive symptoms and those with a score range of 20–27 as having severe depressive symptoms. There were no cases of severe depressive symptoms. Due to the relatively small numbers of respondents with moderately severe depressive symptoms (< 2% of the total), this was added up to those with moderate depressive symptoms.

Financial stress was assessed through the question ‘Have you had problems paying your household bills in the past year?’ This was re-classified into those with and those without problems paying their household bills. Those who had problems paying their household bills were classified as having financial stress. Additionally, a sub-question was asked about how many people the participants were supporting financially with their own wages whereby we especially looked at the number of family members living in Ghana as a source of financial burden from the homeland. Respondents were split into (approximately) equal halves according to the number of people they supported in Ghana. Little less than half of the participants supported ≤ 3 people in Ghana, whilst the rest supported > 3 people. We therefore classified them into two groups, thus those who supported ≤ 3 people and those supporting > 3 people financially in Ghana. Those who provided financial support to > 3 people in Ghana were considered as having financial burden from Ghana.

### Covariates

We also assessed the following variables: adverse health behaviour and body mass index (BMI). Adverse health behaviour was defined as lack of adequate physical activity and smoking. Physical activity was assessed through the question ‘If you add it up, on average, how many days per week do you do at least half an hour of cycling, odd jobs, DIY, gardening or sport?’ Physical activity was re-classified into two categories: ≥ 5 days, 30 min/day versus < 5 days, 30 min/day. Physical activity of less than 5 days, 30 min/day was considered as lack of adequate physical activity. Weight was measured in light clothing to the nearest 0.1 kg.

Height was measured without shoes with a measuring tape to the nearest 0.1 cm. Body mass index (BMI) was calculated as weight in kilogram (kg) divided by height in meter square (m^2^). Overweight was defined as BMI 25–29.9 kg/m^2^ and obesity was defined as BMI ≥ 30 kg/m^2^.

### Data analysis

We combined men and women because of small numbers. Nonetheless, we checked for interactions between sex and the psychosocial stresses and none was statistically significant.

To study associations between psychosocial stress and hypertension, binary logistic regression analysis were performed in which we adjusted for important risk factors for hypertension such as age, sex and BMI. All statistical tests were two-tailed and *P*-values < 0.05 were considered statistically significant. All data analyses were performed using SPSS 18.0 for Windows.

## Results

### Characteristics of the study population

Table 1 shows the characteristics of the study population. Overall, 212 participants were studied. The mean length of stay in the Netherlands was 15.1 years. Nearly 81% of the participants were either overweight or obese. Only 0.9% of the participants smoked whilst 4.6% were ex-smokers. Overall, 56.1% of the participants lacked adequate physical activity. 20.0% of the participants had mild depressive symptoms, whilst 9.3% had moderate depressive symptoms. 34.8% of the respondents had financial stress (problems paying household bills). 52.7% of those supporting people in Ghana experienced financial burden (support > 3 people in Ghana) from the homeland. The overall prevalence of hypertension was 54.7% and 44.8% of the hypertensive individuals were on blood pressure medication. Overall, there were more hypertensive individuals with high systolic blood pressure than those with high diastolic blood pressure (p = 0.00).

**Table 1:** Characteristics of the study population and prevalence of the variables

**(i)**	
	Mean (Std. Deviation)
Age (yrs)	44.6 (8.9)
Height (m)	1.64 (0.08)
Weight (Kg)	78.8 (13.3)
BMI (Kg/m^2^)	29.4 (5.2)
Systolic blood pressure (mmHg)	138.9 (19.7)
Diastolic blood pressure (mmHg)	84.2 (12.2)
Length of stay in Netherlands (years)	15.1 (7.0)
**(ii)**	
	**N (%)**
Sex	
Men	100 (45.2%)
Women	121 (54.8%)
Age groups	
18 – 39	60 (27.1)
40 – 49	88 (39.8)
> 50	73 (33.0)
Education level	
Primary	35 (15.8)
Secondary	116 (52.5)
University/Tertiary	59 (26.7)
Religiousity, yes	218 (98.6)
Christianity	184 (83.2)
Islam	4 (1.8)
Other	31 (14.0)
Hypertension, overall	116 (54.7)
High systolic blood pressure	89 (42.8)
High diastolic blood pressure	56 (27.6)
Anti-hypertensive medication, yes	52 (24.5)
Experienced discrimination	
Low level	73 (33.2)
Moderate	69 (31.4)
High	78 (35.5)
Depressive symptoms	
Minimal	145 (70.7)
Mild	41 (20.0)
Moderate	19 (9.3)
Financial stress, overall	77 (34.8)
Number of people in Ghana supporting financially (¶)	4.5 (3.2)
Financial burden from Ghana, support > 3ppl from GH	77 (52.7)
Smoking	
Current smoker	2 (0.9)
Ex-smoker	10 (4.6)
Lack of adequate PA	124 (56.1)
Overweight	88 (41.5)
Obesity	83 (39.2)

(i) Characteristics of the study population in mean and their standard deviation.

(ii) Further characteristics of the study population and prevalence of the dependent and independent variables and covariates.

PA: physical activity.

BP: blood pressure.

(¶): mean (and standard deviation) of people in Ghana supporting financially.

Support > 3ppl from GH: support more than 3 people in Ghana.

### Relationship between psychosocial stresses and hypertension

**Figure 1:**
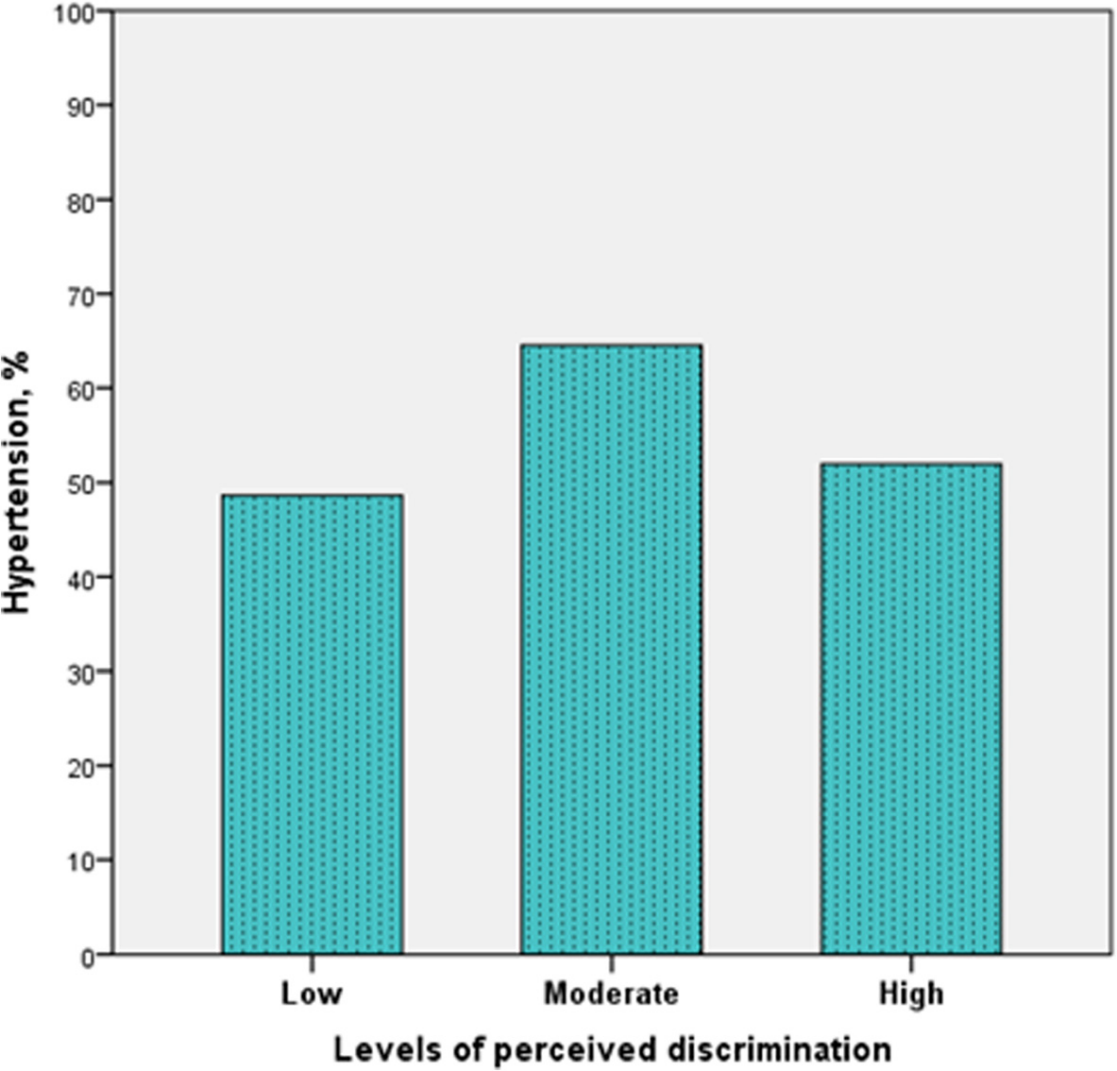
Prevalence of hypertension by perceived discrimination among Ghanaian migrants.

**Figure 2:**
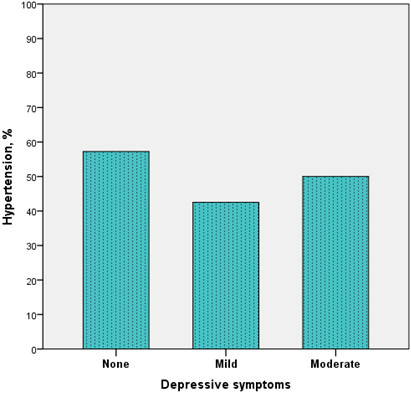
Prevalence of hypertension by depressive symptoms among Ghanaian migrants.

**Figure 3:**
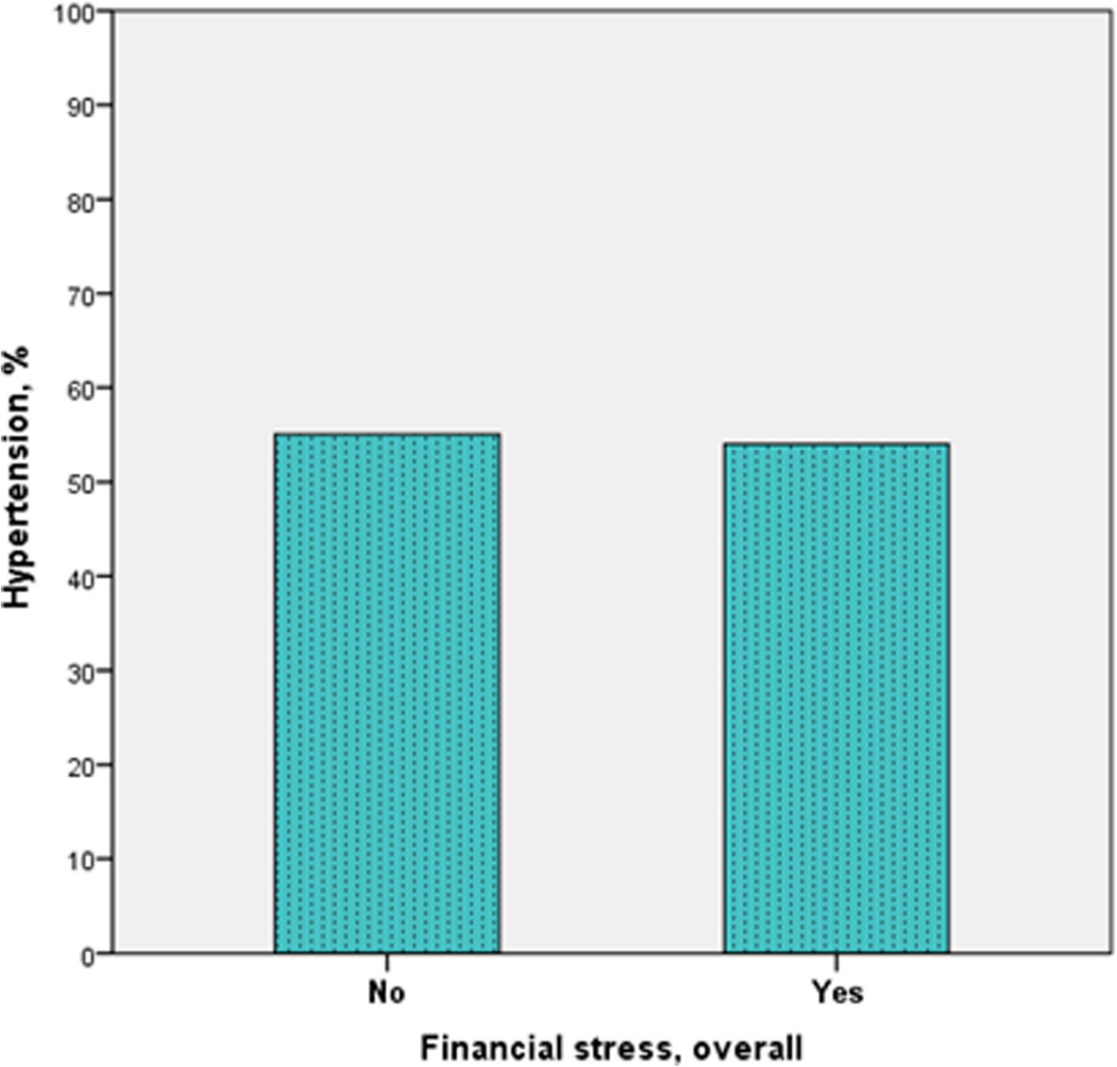
Prevalence of hypertension by overall financial stress among Ghanaian migrants.

**Figure 4:**
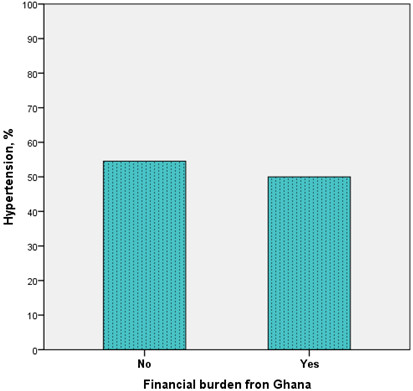
Prevalence of hypertension by financial burden from Ghana among Ghanaian migrants.

Table 2 shows the odds ratios and their corresponding 95% CIs for hypertension by the main independent variables with low level of perceived discrimination and minimal depressive symptoms as reference groups. Neither moderate nor high level of perceived discrimination was strongly associated with hypertension compared with low level of perceived discrimination. There was a suggestion of an association between moderate level of perceived discrimination and hypertension (p = 0.07) (Figure 1). However, this positive association between moderate level of perceived discrimination and hypertension diminished after adjusting for other covariates (sex, age and BMI) (Table 2). Similarly, depressive symptoms as well as financial stress and financial burden from Ghana were also not strongly related to hypertension [Figures 2, 3 and 4 & Table 2].

**Table 2:** Odds ratios hypertension (overall) by psychosocial stress

	**OR (95% CI) Unadjusted**	***p-value***	**OR (95% CI) Adjusted for age, sex & BMI**	***p-value***
**Level of perceived discrimination**				
-Low	1.00		1.00	
-Moderate	1.92 (0.96 – 3.86)	0.07	1.52 (0.70 – 3.33)	0.29
-High	1.14 (0.60 – 2.17)	0.68	0.99 (0.47 – 2.08)	0.99
**Depressive symptoms**				
-Minimal	1.00		1.00	
-Mild	0.55 (0.27 – 1.13)	0.10	0.52 (0.24 – 1.15)	0.11
-Moderate	0.75 (0.28 – 2.00)	0.56	0.81 (0.26 – 2.49)	0.71
**Financial stress, overall**	0.96 (0.54 – 1.69)	0.89	0.71 (0.37 – 1.36)	0.31
**Financial burden from Ghana**	0.83 (0.43 – 1.62)	0.59	0.74 (0.35 – 1.57)	0.44

Low level of perceived discrimination and minimal depressive symptoms are the reference groups.

Table 3 shows the odds ratios and their corresponding 95% CIs for systolic hypertension and diastolic hypertension by the main independent variables excluding individuals on blood pressure medication. None of the main psychosocial stressors included in our study was associated with systolic hypertension or diastolic hypertension.

**Table 3:** Odds ratio of systolic and diastolic hypertension by psychosocial stress

	**Systolic hypertension**				**Diastolic hypertension**			
	**OR (95% CI) unadjusted**	***p-value***	**OR (95% CI) adjusted for age, sex & BMI**	***p-value***	**OR (95% CI) unadjusted**	***p-value***	**OR (95% CI) adjusted for age, sex & BMI**	***p-value***
**Level of perceived discrimination**								
-low	1.00		1.00		1.00		1.00	
-moderate	1.55 (0.66 – 3.60)	0.31	1.46 (0.59 – 3.62)	0.41	1.51 (0.59 – 3.87)	0.40	1.91 (0.66 – 5.54)	0.23
-high	1.46 (0.69 – 3.10)	0.32	1.49 (0.66 – 3.37)	0.34	0.72 (0.29 – 1.83)	0.49	0.87 (0.32 – 2.40)	0.79
**Depressive symptoms**								
-minimal	1.00		1.00		1.00		1.00	
-mild	0.44 (0.18 – 1.07)	0.07	0.50 (0.19 – 1.28)	0.15	0.98 (0.38 – 2.54)	0.96	0.75 (0.25 – 2.19)	0.59
moderate	0.29 (0.06 – 1.38)	0.12	0.32 (0.06 – 1.63)	1.17	0.34 (0.04 – 2.80)	0.32	0.32 (0.04 – 2.96)	0.32
**Financial stress, overall**	0.99 (0.50 –1.96)	0.97	0.88(0.42 – 1.84)	0.73	0.83 (0.36 – 1.90)	0.66	0.78 (0.31 – 1.92)	0.59
**Financial burden from Ghana**	1.08 (0.47 – 2.51)	0.85	0.88 (0.34 – 2.24)	0.79	0.91 (0.32 – 2.58)	0.86	0.93 (0.28 – 3.15)	0.91

Low level of perceived discrimination and minimal depressive symptoms are the reference groups.

## Discussion

Information on the relationship between psychosocial stress and hypertension among recently migrated SSA populations in Western countries is rare. However, psychosocial stress is perceived as an important contributor to hypertension among migrants as shown by our group in a prior study on hypertensive Ghanaian, African-Surinamese and Dutch patients [20,21]. In addition, many Ghanaian and Surinamese respondents in the aforementioned study felt a return to their homeland could even cure hypertension, because their blood pressure is lower when they are in their country of origin. Hence some Ghanaians and Surinamese even discontinue their anti-hypertensive medication use when visiting their homelands. Many other previous reports have linked various psychosocial stressors for example work strain, social environment, emotional distress and or depression to hypertension [28], however the literature has been inconsistent with regard to a strong association between these two [13-15]. As reported in many other studies, we found no evidence for an association between the psychosocial stresses we assessed, namely experienced discrimination, depressive symptoms and financial stress, and hypertension among Ghanaian migrants. Systolic hypertension is more common among the middle-aged and older people than diastolic hypertension [29]. This is reflected in this study as well. However, neither systolic nor diastolic hypertension showed strong association with the psychosocial stresses.

Discrimination has been widely reported to be strongly related to psychosocial stress and some of the ill health conditions associated with psychosocial stresses [28,30], but there are other studies which suggest otherwise. For example, Kaholokula *et al.*[31] reported no strong association between perceived racism and blood pressure among Native Hawaiians after adjusting for potential confounders. Similarly, the Metro Atlanta study [32] reported no significant association between exposure to stress related racial discrimination and prevalence of hypertension among African Americans. As reported in some of these previous studies, we found no clear association between perceived discrimination and hypertension.

While some studies have suggested depression may be more common among hypertensive individuals, the majority have reported no association between hypertension and depression [15]. Our findings support the results reported by the vast majority of studies on this topic, but it needs to be emphasized that our study only assessed depressive symptoms and not depressive disorder according to the DSM-IV mental disorder which make use of much stricter criteria than the one we used. However the PHQ questionnaire used by this study has been shown to be valid in identifying depressive symptoms in a variety of populations, including SSA origin populations [33]. Despite the less strict criteria no significant association could be observed between hypertension and depressive symptoms. It, however, needs to be emphasized that the study was based on organizations within the Ghanaian community and that involvement in an organization may mitigate the effect of stress as these organizations do not only serve religious function but also provide social function for the community [19].

In the relatively new migrant groups, for example Ghanaians who are also known for providing generous financial assistance to their families in Ghana [19], little is known about how financial burden abroad and from homeland impact their lives and health. In their effort to meet their financial obligations here in Europe and from their homeland, some Ghanaians do double jobs on daily basis [19]. This all adds to the work strain which in turn can have an impact on one’s health. In this study, however, we could not observe any significant (cross-sectional) association between financial stress or financial burden from the homeland and hypertension. However, a recent longitudinal study showed that favourable changes in financial strain were associated with reduced ambulatory systolic blood pressure [34]. The reason for the weak relationship in this study is difficult to explain, but some of the important indicators of financial strain were not included in this study. Financial stress was not explicitly asked, instead a single question (having problems with payment of household bills) was used as a measure for having financial stress. Problems with payment of (household) bills are among the indicators of financial hardships, but other indicators such as difficulty purchasing food or finding a sustainable job, lack of essential goods like clothing, facilities and services [35] are equally important. Therefore those who were experiencing other forms of financial strain or stress from financial problems could have been missed. Moreover, social desirability bias may be an issue. There may have been under-reporting of financial stress due to the fact that people may not readily report financial difficulties.

The prevalence of hypertension found among our study group is very high and there is no clear explanation for this. This may, however, be due to a combination of genetic and environmental factors. The latter may play a substantial role as studies in Ghana where the respondents originate from show a lower prevalence (29.4%) of hypertension [7]. For example, we showed in our previous paper [24] that prevalence of hypertension was higher in overweight and obese individuals than in those with normal weight. Also in a previous study, Ghanaians in the Netherlands were ten times more likely than Ghanaians in rural Ghana to be overweight and obese [36]. In this study, the majority of the participants were either overweight or obese (80.7%) and over half of the respondents do not exercise sufficiently (56.1%). These figures are unfavourable compared to the rest of Amsterdam, where 40% is either overweight or obese and 38% do not exercise sufficiently [37]. We therefore assumed that changes in lifestyle and related factors such as increasing obesity and lack of physical activity following migration may play an important role, perhaps more than psychosocial stress, in the high prevalence rate of hypertension among Ghanaians in Amsterdam. Moreover, poor early life circumstances such as poor nutrition during foetal life and infancy might play a role in the high prevalence of hypertension among this group. The majority of studies on this topic have associated malnutrition during foetal life or infancy with risk of high blood pressure in later life [38].

### Strengths and limitations

There are limitations and strengths to this study. First, it is a cross-sectional study with a relatively small sample size. However, this is one of the largest studies among one homogenous SSA group living in a Western country. Earlier studies were compelled to combined heterogeneous SSA groups because of smaller numbers [3,4]. Also, we did not assess the cortisol production, an important stress parameter, among the participants in this study. The blood pressure levels we used were based on the average measurements at a single visit, which might have overestimated the prevalence rates. In addition, we had no data on perceived social support. Like discrimination and financial problems, lack of social support may place people at risk of psychosocial stress. Moreover, selection bias may be an issue as the study was based on organizations within the Ghanaian community. Therefore Ghanaians who were not affiliated with these organizations might have been missed. Nevertheless, evidence suggests that most Ghanaians in the Netherlands are affiliated with Ghanaian organizations [19]. We therefore expect that our study is representative of the Ghanaian population living in Amsterdam. Moreover the participants were a mix from different religious and education backgrounds (including Christians and Muslims, low and high educated) and different age groups. This enhances the generalizability of our study findings to other Ghanaian migrant communities.

## Conclusions

In conclusion, we found no evidence that psychosocial stress was associated with hypertension among this recently migrated SSA population. More efforts are needed to unravel other potential factors that may underlie the high prevalence of hypertension among these populations.

## Competing interests

The authors declare that they have no competing interests.

## Authors’ contributions

CA and MN conceived the study, and participated in its design and coordination and helped to draft the manuscript. BA performed the statistical analysis and drafted the manuscript with the support of CA. LB, BJB and HD participated in the design of the study and helped to draft the manuscript. All authors read and approved the final manuscript.

## Pre-publication history

The pre-publication history for this paper can be accessed here:

http://www.biomedcentral.com/1471-2458/14/692/prepub
